# Design of a Machine Learning-Based Intelligent Middleware Platform for a Heterogeneous Private Edge Cloud System

**DOI:** 10.3390/s21227701

**Published:** 2021-11-19

**Authors:** Sayed-Chhattan Shah

**Affiliations:** Mobile Grid and Cloud Computing Lab, Department of Information Communication Engineering, Hankuk University of Foreign Studies, Seoul 02450, Korea; chhattanshah@ieee.org

**Keywords:** fog computing, edge computing, resource management, intelligent network layer, local cluster heterogeneous network, internet of things applications, mobile ad hoc network

## Abstract

Recent advances in mobile technologies have facilitated the development of a new class of smart city and fifth-generation (5G) network applications. These applications have diverse requirements, such as low latencies, high data rates, significant amounts of computing and storage resources, and access to sensors and actuators. A heterogeneous private edge cloud system was proposed to address the requirements of these applications. The proposed heterogeneous private edge cloud system is characterized by a complex and dynamic multilayer network and computing infrastructure. Efficient management and utilization of this infrastructure may increase data rates and reduce data latency, data privacy risks, and traffic to the core Internet network. A novel intelligent middleware platform is proposed in the current study to manage and utilize heterogeneous private edge cloud infrastructure efficiently. The proposed platform aims to provide computing, data collection, and data storage services to support emerging resource-intensive and non-resource-intensive smart city and 5G network applications. It aims to leverage regression analysis and reinforcement learning methods to solve the problem of efficiently allocating heterogeneous resources to application tasks. This platform adopts parallel transmission techniques, dynamic interface allocation techniques, and machine learning-based algorithms in a dynamic multilayer network infrastructure to improve network and application performance. Moreover, it uses container and device virtualization technologies to address problems related to heterogeneous hardware and execution environments.

## 1. Introduction

Recent advances in mobile technologies have enabled a new class of smart city and fifth-generation (5G) network applications, such as smart homes and real-time situation analyses. These applications have diverse requirements, such as low latencies, high data rates, significant amounts of computing and storage resources, and access to the Internet of Things (IoT) devices. To address the requirements of these applications, several edge computing systems, such as cloudlet computing, mobile edge computing, and fog computing, were proposed [1,2,3,4]. The deployment of edge computing systems requires the addition of new infrastructure or the updating of existing infrastructure. Edge computing systems also do not utilize the capabilities of end devices, such as smartphones, mobile robots, and smart vehicles, which are equipped with multicore central and graphical processing units, several sensors, or multiple wireless communication technologies. A heterogeneous private edge cloud system is proposed to overcome the drawbacks of edge computing systems.

A heterogeneous private edge cloud system [1] is a small-scale cloud data center in a local physical area, such as a house or an office. It consists of various stationary and mobile devices, such as personal computers, mobile robots, smartphones, and sensors, which are interconnected through single or multiple infrastructure-based or infrastructure-less wireless local area networks (LANs). A heterogeneous private edge cloud system is characterized by a complex and dynamic multilayer network and computing infrastructure. The efficient management and utilization of this infrastructure may increase data rates and reduce data latency, data privacy risks, and traffic to the core Internet network. 

A middleware platform is required to manage and utilize the heterogeneous private edge cloud system infrastructure efficiently. Such a platform is responsible for the discovery, monitoring, allocation, and management of resources. Compared with those of conventional systems, such as mobile ad hoc clouds [2] or edge clouds [4], the design of a middleware platform for a heterogeneous private edge cloud system is more challenging due to (1) the presence of heterogeneous devices, such as sensors, smartphones, mobile robots, and personal computers, and (2) a multilayer network environment. Existing middleware platforms are divided into two categories: middleware platforms for IoT systems and middleware platforms for distributed computing systems. Middleware platforms for IoT systems provide access to and control physical devices. They also support data collection, data analysis, or application composition services. These platforms, however, do not provide computing services, do not utilize network-level information, such as link quality and link lifetime, and are not designed for complex multilayer network environments. Most middleware platforms for distributed computing systems provide computing or storage services but do not support data collection and actuation services; they are also not designed for heterogeneous multilayer network environments. Therefore, they do not efficiently utilize heterogeneous routes, simultaneous transmission on multiple communication technologies, and several network-level parameters, such as link quality and lifetimes.

The current study presents the design of an intelligent middleware platform that aims to utilize the characteristics efficiently and address the challenges of heterogeneous computing and a multilayer network environment to (1) manage heterogeneous computing and network resources efficiently, and (2) provide task processing, data collection, and data storage services to support emerging smart city and 5G network applications. The new platform consists of two layers: a software-defined network (SDN) and a machine learning-based multi-network management layer and a machine learning-based resource management layer. The multi-network management layer aims to (i) use the capabilities of machine learning and SDN to improve network and application performance, (ii) provide serial and parallel data transmission services across multiple heterogeneous networks, (iii) support the dynamic allocation of network interfaces, and (iv) adopt new machine learning-based link quality and Markov chain-based link lifetime estimation techniques to reduce communication and energy consumption costs. The resource management layer aims to (i) leverage regression analysis and reinforcement learning methods to allocate heterogeneous computing and network resources efficiently to application tasks and (ii) use parallel transmission techniques, dynamic interface allocation techniques, and network-level parameters to address diverse application requirements.

This paper is organized as follows. Section 2 describes motivating smart city and 5G network applications. Section 3 describes the role of heterogeneous private edge cloud in a cloud and edge computing ecosystem. The key characteristics and challenges of a heterogeneous private edge cloud system are described in Section 4. Section 5 presents a detailed overview and comparison of existing middleware platforms for IoT systems and distributed computing systems. Section 6 presents the design of a new intelligent middleware platform for a heterogeneous private edge cloud system. Section 7 concludes the study.

## 2. Motivating Applications and Use Cases

Smart city and 5G network applications can be divided into two major categories: non-resource-intensive and resource-intensive applications. Both categories are further divided into two subcategories: non-real-time and real-time. Latency and data rate requirements of real-time and non-real-time smart city and 5G use cases are given in Table 1. Examples of motivating applications and use cases are briefly described as follows. 

### 2.1. Smart Home 

The smart home application provides home and health management services. It uses surveillance cameras, audio sensors, smart grid sensors, and bio-sensors for collecting environment data and the health data of individuals at home. Then, the application uses computationally intensive machine learning models and audio-video processing algorithms for activity and situation recognition, early threat detection, emotion detection, and abnormal health condition detection. On the basis of the analysis, the application takes necessary actions, such as sending situation information to an emergency or security service provider or asking a mobile robot for help.

### 2.2. Real-Time Situation Analysis

Real-time situation analysis is also a computationally intensive real-time application. It requires access to video surveillance cameras in a target area to collect video data, vast amounts of computing and storage resources to execute computationally intensive video analysis tasks for situation analysis, and actuators to perform necessary actions. Video sensors are assumed to be either deployed in the target area or mobile robots or micro-drones equipped with video sensors are rented for data collection.

### 2.3. Training of Machine Learning Model Use Case

The training and inference of machine learning models for computer vision and language modeling are extremely processing-intensive tasks that require an enormous amount of computing power.

### 2.4. Big Data Analysis Use Case

Devices used in various smart city sectors [1], such as transportation, healthcare, and agriculture, generate a huge amount of data. The appropriate understating of these data presents numerous opportunities to organizations and governments. However, the storage and real-time analysis of a vast amount of data present a huge challenge.

Applications and use cases, such as smart homes and real-time situation analyses, require low data latencies, high data rates, vast amounts of computing and storage resources, and access to sensors and actuators. The conventional cloud computing systems are unable to fulfill the requirements of these applications [1,4,9]. The middleware platform for a heterogeneous private edge cloud system aims to fulfill the requirements of these applications.

## 3. The Role of Heterogeneous Private Edge Cloud System

**Figure 1:**
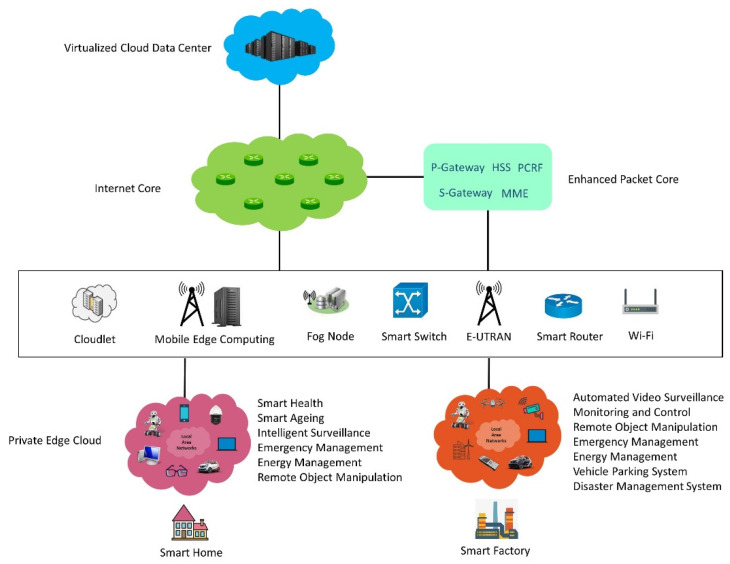
The role of heterogeneous private edge clouds in a cloud and edge computing ecosystem with examples of applications that may be considered at smart homes, smart factories, and smart buildings [1].

The role of heterogeneous private edge cloud [1] in a cloud and edge computing ecosystem is depicted in Figure 1. Several stationary and mobile devices such as personal computers, mobile robots, smartphones, and sensors available within a local area are combined to create a small-scale cloud data center. The heterogeneous private edge cloud may provide several services such as computing, data storage, data processing, and data caching. The private edge cloud can be connected to classical edge computing systems such as a mobile edge computing system or fog computing system, or it may be connected to a central virtualized cloud data center on the Internet via long-term evolution (LTE) or 5G networks.

## 4. Characteristics and Challenges of a Heterogeneous Private Edge Cloud System

A heterogeneous private edge cloud system integrates several computing and networking technologies, such as cloud computing, mobile computing, edge computing, mobile ad hoc networking, and infrastructure-based local area networking [1]. Such integration provides numerous beneficial characteristics but also poses challenges. The key characteristics and challenges are described as follows. 

### 4.1. Heterogeneous Computing Resources

A heterogeneous private edge cloud includes numerous heterogeneous devices, such as personal computers, mobile robots, smart cars, smartphones, and sensors. These devices differ in terms of system architecture, operating system, execution environment, and speed. A device may offer a single service or a multitude of services, such as data collection, task execution, data storage, and data caching. Such an environment introduces numerous challenges, as follows:

- Uniform representation and control of heterogeneous devices;
- Efficient discovery, registration, and monitoring of a wide range of devices and services;
- Allocation of heterogeneous computing, sensing, and actuating resources to emerging application tasks with a diverse quality of service requirements;
- Execution or processing of tasks submitted by another homogenous or heterogeneous device; and
- Communication and collaboration of heterogeneous services regardless of application platforms, programming languages, operating systems, or system architecture.

### 4.2. Heterogeneous and Multilayer Communication and Network Infrastructure

A heterogeneous private edge cloud consists of stationary and mobile devices. Devices equipped with multiple wireless communication technologies are common, and they will become more persistent with the deployment of 5G networks. Wireless communication technologies have diverse features, and they also differ in terms of bandwidth, latency, energy consumption, communication range, reliability, and network topology. Devices may communicate via infrastructure-based wireless LAN technologies, infrastructure-less wireless LAN technologies, or both. These characteristics enable the creation of a communication and network infrastructure with multiple and overlapping communication topologies, diverse source-to-destination links, and dynamic topologies and links due to the existence of mobile nodes.

Given the aforementioned characteristics, heterogeneous and multilayer communication and network infrastructure also introduce several challenges, as follows:

- Development of communication and energy consumption cost estimation models and link quality and link lifetime estimation models for heterogeneous wireless communication technologies;
- Design of efficient discovery and monitoring protocol or set of protocols for a multilayer network infrastructure that is characterized by multiple and overlapping communication topologies, diverse source-to-destination links, and dynamic topologies and links;
- Development of routing and network management protocols capable of selecting routes and supporting data transmission services based on a diverse quality of service requirements over a multilayer network infrastructure; and
- Design of a network layer that provides a unified and easy-to-use interface to higher layers.

## 5. State of the Art

Middleware platforms are divided into two categories: middleware platforms for IoT systems and middleware platforms for distributed computing systems. This section is divided into subsections. Section 5.1 describes middleware platforms for IoT systems, whereas Section 5.2 describes middleware platforms for distributed computing systems. 

### 5.1. Middleware Platforms for IoT Systems

Middleware platforms for IoT systems provide access to and control physical devices. They also support data collection, data analysis, or application composition services. These platforms, however, do not provide computing services, do not utilize network-level information, such as link quality and link lifetime, and are not designed for complex multilayer network environments.

For example, Hydra [10] platform enables applications to access heterogeneous physical devices, supports multiple communication technologies, and uses web service technology to address heterogeneity-related problems. Global Sensor Networks [11] platform virtualize sensors to address device heterogeneity. Google Fit [12] provides a cloud storage service to store data from heterogeneous IoT devices and applications, and native support for Bluetooth low energy. Xively [13] is a cloud-based platform that provides data collection and storage services whereas Calvin [14] and Node-RED [15] support the composition and management of IoT applications. MQTT is a lightweight message transport protocol based on a publish-subscribe model [16]. It enables heterogeneous devices and applications to communicate with another and provides a reliable and secure message delivery service. An IOcloud proposed in [17] uses IoT nodes as active elements of infrastructure. IoT node is defined as any computing unit such as smartphone and Raspberry Pi that host IoT resources such as LED or temperature sensor. A high-level design of a message-service-oriented middleware for the fog of things paradigm is given in [18]. The proposed middleware enables devices to exchange IoT data through messages and supports the migration of services when a gateway or a fog node fails. The middleware is distributed among several fog of things entities such as fog servers and fog gateways, and it is based on a Microservices architecture. VIRTUS middleware based on the publish-subscribe communication model is proposed in [19]. It is designed for healthcare applications and aims to provide real-time and secure communication among heterogeneous devices. A data-centric middleware based on a publish-subscribe communication model is proposed in [20]. The middleware is designed for a dynamic mobile environment. It addresses frequent connection and disconnection-related issues and also supports various quality of service (QoS) levels. A location and activity-aware mobile distributed sensing platform is proposed in [21]. The platform enables users to collect sensor data on-demand based on user requirements and location. It uses sensing as a service model and a concept of a virtual sensor. Any device which produces data can be a virtual sensor. The platform consists of three components: Context-aware Mobile Sensor Data Engine, Activity-aware Module, and Location-aware Module. Context-aware Mobile Sensor Data Engine enables sensor data collection and processing without any coding efforts. It supports push and pull data streaming mechanisms as well as decentralized and centralized communication. The activity-aware module can recognize several activities such as biking, walking, and running. The location-aware module is able to recognize when a user enters or leaves a certain area. A systematic survey of several IoT platforms is presented in [22]. Most of these platforms support heterogeneous sensing and actuation devices and several standard communication paradigms and protocols. To address interoperability, either a gateway is used or devices are required to support standard or commonly used protocols such as HTTP or MQTT. 

### 5.2. Middleware Platforms for Distributed Computing Systems

Most middleware platforms for distributed computing systems, such as edge clouds [5] and mobile ad hoc clouds [4,6], provide computing or storage services but do not support data collection and actuation services; they are also not designed for heterogeneous multilayer network environments. Therefore, they do not efficiently utilize heterogeneous routes, simultaneous transmission on multiple communication technologies, and several network-level parameters, such as link quality and lifetimes. Most of these platforms do not use end devices as service provider nodes.

For example, Hyrax [23] platform supports cloud computing on android mobile devices. Fram [24] is a content distribution middleware system for android devices that is designed for an ad hoc environment and also addresses node mobility. Femto Clouds [25] is another platform that enables nearby devices to execute parallel tasks. Devices in Femto Clouds communicate via Wi-Fi technology. Multipeer Connectivity [26] is a framework that enables nearby devices to communicate via messages, stream data, or files. It uses Wi-Fi, Bluetooth, and Ethernet for underlying communication. A framework proposed in [27] enables mobile devices to communicate with each other via short-range wireless communication technologies such as Wi-Fi and Bluetooth. It introduces the concept of micronet and macronet. A micronet consists of devices connected using a single communication technology whereas macronet is defined as a set of micronets connected through member devices. It uses a store-carry-and-forward communication paradigm for intra-macronet and inter-macronet communication. An OpenStack-based middleware platform is proposed in [28] to manage resources at edge, fog, and cloud levels. In the proposed system, edge devices establish a local area cluster at the edge level to reduce data transfers over public networks. This also reduces data transfer times and thus improves application performance. If local clusters are unable to fulfill application or system requirements, fog level and cloud level resources are used. A middleware to support the execution of crowdsourcing applications on edge cloud is described in [29]. It consists of three layers: link layer, network layer, and service layer. The link layer provides access to several communication technologies such as Wi-Fi and BLE. It supports device discovery and connection operations. The network layer is responsible for routing and network management, and the service layer offers computing, storage, and messaging services. A resource management middleware for the fog-edge environment to fulfill latency requirements of internet of things applications is proposed in [30]. The proposed middleware decides whether to execute a task locally or remotely on a fog server based on network conditions. The middleware consists of several components. Latency estimator component estimates latency which is made up of three parts: last-hop latency to a wireless access point, wide area network latency to reach fog server, and task execution time on the fog server. To estimate last-hop latency, a database is used that stores network latencies for different locations and times. The latency is then estimated by querying this database. For a selection of a fog server to execute an application task, several machine learning models are used.

A resource allocation scheme is proposed in [31] to improve energy efficiency. The proposed scheme focuses on allocation of interdependent tasks and uses transmission power control mechanism. A resource management system is proposed in [32] to improve the response time of latency-sensitive applications. The proposed system makes decisions based on the network, compute, and reliability characteristics of edge nodes. A live video streaming service was used to demonstrate the performance of the system. In [33] a resource allocator named Justice is proposed for cluster managers. The Justice uses deadline information of a job and historical job execution times to improve deadline satisfaction and fairness. A design of a distributed resource allocator for a multi-cloudlet environment is discussed in [34]. The allocator can offload jobs to remote cloud or cloudlets. It is adaptive to the dynamic environment, preserves fairness, and aims to satisfy the requirements of deadlines-oriented applications. It uses job execution times history, local and remote cloudlets utilization information to decide whether the application should run on the current cloudlet, a neighbor cloudlet, or a remote cloud. An online statistical model is used to estimate the resources required to complete a job. Authors of [35,36,37] have investigated the problem of offloading tasks from cloudlets to a remote cloud. The scheme in [37] aims to optimize offloading decisions whereas the scheme in [38] focuses on user fairness. The scheme in [35] aims to decrease execution latency and energy consumption in a multi-cloudlet environment. A feature-wise comparison of the most relevant resource allocation and offloading schemes is shown in Table 2. 

Middlewares for the cloud of things are surveyed in [39]. Authors have (a) discussed numerous features and characteristics of middlewares such as interoperability, context management, and reusability, (b) compared middlewares based on architectures such as distributed, component-based, and service-based, and (c) have discussed numerous challenges and research directions. The survey focuses on high-level features and characteristics. A detailed discussion of resource management algorithms and network management protocols has not been included. A cluster consisting of more than three hundred Raspberry PIs is developed in [40]. Authors in [41] implemented container and cluster technology on single-board computers such as Raspberry PIs. A container-based cluster architecture is proposed. The key elements of the architecture are devices, containers, links, and services. A cluster consists of several devices. Each device hosts containers and each container include services. Containers communicate with one another via links. 

Numerous edge and fog computing architectures and offloading strategies in fog environments are investigated in [42]. Fog computing systems for augmented reality applications are studied in [43]. Authors have investigated several multilayer edge and fog computing architectures, energy optimization, and latency optimization techniques, and offloading approaches. Several approaches described in the study encode regions of interest or transmit compressed data to reduce communication latency. An edge computing-based IoT architecture that uses Microservices and container technology is proposed in [44]. The edge computing layer of the architecture is coupled with AI hardware to meet the requirements of artificial intelligence IoT applications. The architecture consists of an application layer, network layer, perception layer, and newly proposed AI accelerated Microservices layer, which preprocesses the data and provides real-time response to the perception layer. In [45,46,47] an approach was proposed to offload augmented reality application tasks to edge computing systems such as cloudlets. A scheme in [45] also supports offloading of application tasks to an ad hoc cloudlet system which consists of mobile phones and laptops connected via a local area network. A scheme in [47] uses a three-layer architecture to reduce processing delays and energy consumption of mobile augmented reality applications. The architecture consists of the user layer, edge layer, and cloud layer. The edge layer includes three modules: communication, operation platform, and virtualized controller. The communication module provides data transmission services and the operation platform process the offloaded tasks. Virtualized controllers manage network activities, allocate resources to application tasks, and provide storage services. In [48,49] authors have used fog computing systems to train machine learning algorithms. A vehicle-to-edge architecture for augmented reality applications is proposed in [50]. The architecture has three layers: device layer, access network layer, and core network layer. The device layer consists of devices that transfer data. The edge servers are located at access network and core network layers. Edge servers at the access network provide compute and cache services to latency-sensitive applications whereas edge servers at the core network are for delay-tolerant applications. A design of a simple middleware platform for a fog and cloud environment is described in [51]. The proposed middleware addresses the services selection problem by applying fuzzy similarity and TOPSIS (Technique for Order of Preference by Similarity to Ideal Solution) methods. 

A software-defined network (SDN) based architecture for tactical mobile ad hoc network is proposed in [52]. The architecture includes local SDN controllers and global SDN controllers. Local SDN controllers may have a full or partial view of the network and are responsible to collect and send network state information to the global SDN Controller, which constructs a global view of the network and send control information to forwarding nodes through a local SDN controller. An SDN-enabled architecture for a mobile ad hoc network is proposed in [53]. A centralized SDN controller is deployed on a mobile node in an ad hoc network. Authors have also proposed two zero-control-packet routing protocols for lightweight devices used in the industrial internet of things (IIOT) and three general-purpose centralized routing protocols.

Recently, several machine learning-based schemes were proposed to address routing and resource allocation problems. The scheme developed in [54] uses a machine learning technique to predict link quality, while the schemes presented in [55,56] adopt a deep learning model that uses traffic patterns in a router to predict the next node in the routing path. The scheme proposed in [57] uses a nonlinear regression technique to estimate link quality, while the scheme established in [58] uses machine learning to improve multi-hop wireless routing. To manage resources in a distributed computing environment, a reinforcement learning-based approach was developed in [59]. In [60], reinforcement learning was applied to reduce application execution times. A link lifetime prediction scheme was proposed in [61]. Existing machine learning-based algorithms and schemes are also not designed for a multilayer LAN infrastructure, and therefore, they do not utilize a vast amount of data generated at the network.

A comparison of the proposed middleware platform with existing platforms is provided in Table 3. The heterogeneous private edge cloud system is based on our previous project “mobile ad hoc cloud” [4], in which multiple mobile devices interconnected through a mobile ad hoc network are combined to create a virtual super-computing node. The key differences between a mobile ad hoc cloud and a heterogeneous private edge cloud system were discussed in [1].

## 6. Design of an Intelligent Middleware Platform

This section presents the design of a new intelligent middleware platform for a heterogeneous private edge cloud system. The proposed middleware platform aims to fulfill the following requirements: (a) efficient discovery, registration, and monitoring of a wide range of services and devices interconnected through the multi-layer network infrastructure, (b) efficient and robust allocation of heterogeneous computing, sensing, and actuating resources to emerging application tasks with a diverse quality of service requirements, (c) communication and collaboration of heterogeneous devices and services regardless of application platforms, programming languages, operating systems, or system architecture, (d) efficient and reliable routing and network management protocols capable of selecting routes and supporting data transmission services based on a diverse quality of service requirements over the multi-layer network infrastructure, and (e) unified and easy to use interface to higher layers. 

**Figure 2:**
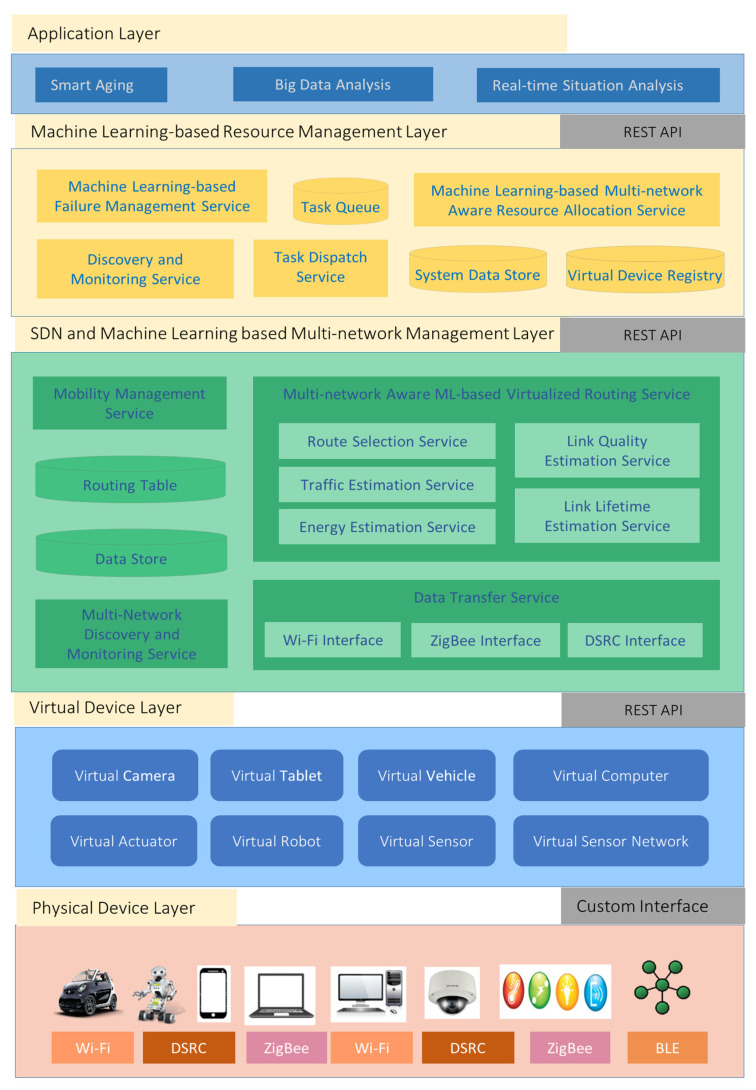
Design of an intelligent middleware platform.

The proposed middleware platform comprises four layers. Each layer includes several components. The design of an intelligent middleware platform is presented in Figure 2. 

### 6.1. Physical Device Layer

This layer includes devices, such as sensors, actuators, personal computers, and smartphones. Sensors provide data collection services, and actuators provide device movement and control services. High-end devices, such as personal computers and smartphones, provide task processing and storage services. A single device can provide multiple services. For example, a smartphone may provide task processing and data storage services, and a mobile robot equipped with sensors may provide data collection, data storage, task processing, and actuation services.

Devices that provide task processing services are assumed to host a container engine. Container technology, such as Docker, enables virtualization at the operating system level and is used to address problems related to a heterogeneous operating system and execution environments. Containers are more lightweight than conventional virtual machines; thus, they are also supported on constrained devices.

### 6.2. Virtual Device Layer

**Figure 3:**
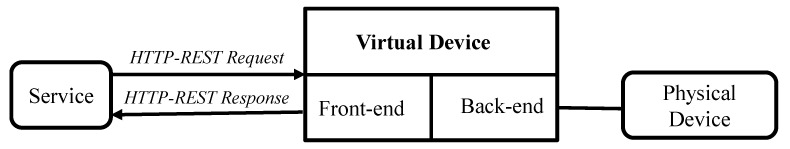
Block diagram of a virtual device.

This layer consists of virtual devices. A virtual device is a semantically and functionally enriched representation of a physical device. It uses web technology to provide a uniform interface to other devices and services. A block diagram of a virtual device is presented in Figure 3. A virtual device consists of front-end and back-end modules. The front-end module enables applications or services to access resources or services provided by a physical device via a standard web interface. The back-end module communicates with physical devices via device-specific protocols and mechanisms.

A single virtual device may also represent multiple physical devices. For example, a virtual device may represent a sensor network that monitors a specific area and a smartphone that includes sensors. Virtual devices are divided into two categories: simple and container-based virtual devices.

**Figure 4:**
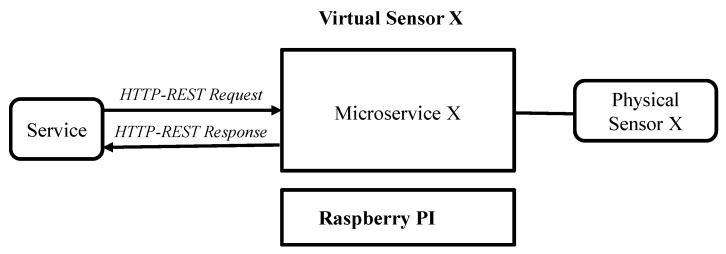
Implementation of a virtual device.

Simple virtual devices, such as virtual sensors or actuators, provide data collection or actuation services. A microservice can be used to implement a simple virtual device. A microservice or simple virtual device either runs on the physical device that it represents or on another physical device, such as an RPi or WiFi router. Figure 4 shows a Physical Sensor X is represented by a Virtual Sensor X hosted on a Raspberry PI computer. A microservice X is used to implement Virtual Sensor X. 

**Figure 5:**
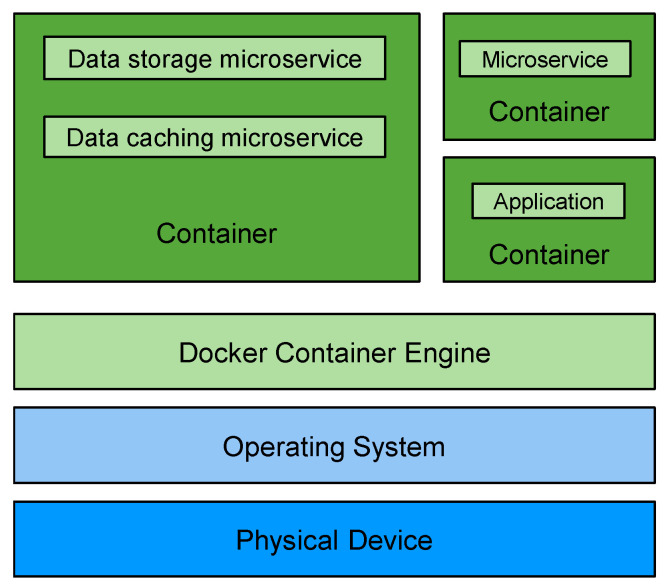
Container-based physical device.

A container-based virtual device represents a high-end physical device that provides task processing, data caching, or data storage services. A container-based virtual device hosts a container engine that executes containerized applications or microservices. Container technology [4] at the virtual device layer is used to address interoperability requirements. The architecture of a container-based physical device that also hosts containerized microservices is illustrated in Figure 5. 

### 6.3. SDN and Machine Learning-Based Multi-Network Management Layer

A multi-network infrastructure generates a vast amount of network data. The management of a multi-network infrastructure and the utilization of network data to maximize system performance are challenging tasks [55]. Existing network-level protocols are not designed for a heterogeneous multi-network infrastructure that integrates ad hoc and infrastructure-based network technologies. Therefore, they do not efficiently utilize heterogeneous routes, simultaneous transmission on multiple communication technologies, and several network-level parameters, such as link quality, link lifetime, and transmission energy consumption. Recently, several machine learning-based protocols were proposed to address network management and routing problems. However, these protocols are designed for either infrastructure-based or ad hoc networks. Thus, they use conventional attributes, such as throughput, and are unable to utilize a vast amount of data generated by a complex heterogeneous multi-network infrastructure. 

A new SDN and machine learning-based multi-network management layer aim to use the capabilities of machine learning and SDN to enable adaptive, efficient, and reliable communication among devices interconnected via multiple heterogeneous mobile ad hoc and infrastructure-based LANs. This layer comprises eight services.

***Data Transfer Service:*** This service aims to enable efficient and reliable transmission of data across multiple heterogeneous networks. It provides a simple and unified interface to network layer services and can interface with multiple communication technologies, such as Wi-Fi and Bluetooth Low Energy (BLE), via technology-specific protocols. The functions of this service include the following: (1) packing and unpacking of data, (2) transmission of data on a route selected by a routing service or simultaneous transmission of data on multiple routes via multiple communication technologies, and (3) communication with multiple technologies to transmit and receive data. 

***Multi-Network Discovery and Monitoring Service:*** This service aims to discover and monitor information about multi-network infrastructure. The collected information is stored in a semantically enriched data store. Information, such as throughput, delay, energy consumption, and packet loss rate, stored in the semantically enriched data store can be used to train machine learning models [56,57] that predict device and task failures, identify reliable nodes, and optimize network and application performance. 

***Mobility Management Service:*** This service is responsible for maintaining mobility information and using Markov chain-based models to predict the next probable location and time that a device spends at the location. This information can be used to predict link failures and a device reliability index. The design and implementation of the Markov chain-based location prediction mechanism were discussed in [2]. 

***Multi-Network Routing Table:*** A routing table is responsible for storing available routes and characteristics, such as lifetime, energy consumption rate, and available throughput of each route. Raw and historical data are stored in the semantically enriched data store. The routing table proposed in [61] can be extended to support a multi-network environment.

***Multi-Network Aware Machine Learning-Based Virtualized Routing Service:*** This service is responsible for selecting a route or set of routes based on an application’s quality of service (QoS) requirements. It includes the following services. 

***New traffic estimation service:*** The prediction of traffic volume is important in congestion control, resource allocation, and routing [60]. However, measuring traffic volume by using conventional methods is expensive and communication-intensive. This service is responsible for using a new machine learning model to measure network traffic volume.

***Link quality estimation service:*** An accurate estimation of link quality is essential for reliable communication. A link quality estimation service is responsible for using an online machine learning algorithm, such as that presented in [58], to predict link quality on the basis of network-level parameters, such as throughput, packet loss rate, and traffic volume.

***Link lifetime estimation service:*** Link lifetime is crucial for communication performance and energy efficiency. Link lifetime estimation service is responsible for estimating link lifetime on the basis of mobility history and network-level parameters, such as signal strength. The mobility history-based link lifetime estimation model proposed in [2] can be adopted. This model only considers the history of user’s visited locations. To improve its performance, the model can be extended to consider the location and time that a node spends at the location. 

***Link energy estimation service:*** This service aims to use a new machine learning-based model similar to that proposed in [58] to estimate link energy consumption. Compared with existing models, the new model should consider mobility history, link quality, number of dropped and lost packets, signal strength, and quantity of data transmission. 

***Route selection service:*** A centralized SDN-based route selection service is responsible for the selection of single or multiple routes for data transmission on the basis of the application’s QoS requirements. In the case of multiple routes, the service is also responsible for determining the amount of data that should be transmitted to each route. Reinforcement learning-based systems or services are used to address decision-making problems. The route selection service should adopt a reinforcement learning-based model, such as that presented in [60], to select routes. Route selection decisions should be based on the application’s QoS requirements, link quality, link lifetime, and link energy consumption parameters. 

### 6.4. Machine Learning-Based Resource Management Layer 

Existing resource management platforms are divided into two categories. (1) Resource management platforms for IoT systems provide access to and control of physical devices. They also support data collection, data analysis, and application composition services. However, they do not provide computing services and are not designed for complex multilayer network infrastructure. (2) Resource management platforms for distributed computing systems provide computing and storage services. However, they do not focus on data collection and actuation services. Moreover, they are not designed for heterogeneous multilayer network infrastructure, and therefore, are not aware of static and dynamic routes available in each network layer. Consequently, they are unable to fulfill the low latency and high data rate requirements of several smart city and 5G network applications. Existing resource management schemes also use conventional approximate or heuristic algorithms, which are computationally expensive, incur significant overhead, and do not perform well with an increasing number of parameters. Recently, several machine learning techniques, such as those presented in [59,60], were used to address resource management problems. However, these techniques use only a few system-level parameters and they also do not utilize a vast amount of data generated by multi-network infrastructure. A detailed analysis of resource management schemes was provided in [2,31].

A new resource management layer aims to leverage regression analysis and reinforcement learning methods to allocate and manage heterogeneous computing, sensing, actuating, and network resources efficiently. This layer comprises six services and storage units.

***Efficient Discovery and Monitoring Service:*** This service is responsible for discovering and monitoring a wide range of devices and services across multiple heterogeneous networks and for performing the registration and de-registration of devices. The information collected by this service should be used to train machine learning algorithms and make resource management decisions. A similar discovery and monitoring service was developed in [2] for a homogenous network environment.

***System Data Store:*** This service is responsible for storing a large amount of historical application and system-level data, such as application submission time, application completion time, task completion success rate, device failure rate, task completion rate of devices, and energy consumption profile of devices. The data should be used by machine learning algorithms to analyze system performance and predict device and task failures [15]. 

***Task Queue:*** This service is responsible for maintaining a list of tasks submitted by a user and storing task-related information, such as task size, input data size, output data size, task deadline, and data latency requirements. Once a task is completely executed, it is removed from the queue and its information is stored in the system data store. 

***Virtual Device Registry:*** Virtual device registry is responsible for storing a list of available virtual devices. For each device, device-related information, such as speed, energy consumption rate, list and description of services, and communication technologies, is listed. This service is also responsible for storing information required to access the other services.

***Task Dispatch Service:*** This service is responsible for sending tasks and required data to a node selected by a resource allocation service.

***Resource Allocation Service:*** The allocation of resources is a complex and difficult task [2]. A multi-network environment and varied QoS requirements make the process even more difficult [1]. Existing approximate or heuristic schemes are designed to utilize single wireless communication technology or rely on an eclectic system that exclusively selects one distinct communication technology. They also do not efficiently utilize network-level parameters, such as link lifetime and energy consumption. Moreover, they do not perform well with an increasing number of parameters and continuously changing heterogeneous environments. 

The resource allocation service should consider large numbers of the network- and system-level parameters, utilize network-level services, such as dynamic interface allocations and parallel transmissions, and use machine learning-based algorithms, such as those presented in [59,60] to satisfy the requirements of emerging resource-intensive and non-resource-intensive smart city and 5G network applications and improve resource utilization and energy efficiency. 

***Failure Management Service:*** This service is responsible for using machine learning models, such as that adopted in [31], to predict the failure probability of devices and tasks on the basis of the historical device, task, and task assignment information. 

## 7. Conclusions

A heterogeneous private edge cloud system is a small-scale cloud data center in a local physical area, such as a house or an office. It consists of various stationary and mobile devices, such as personal computers, mobile robots, smartphones, and sensors, interconnected through single or multiple infrastructure-based or infrastructure-less wireless LANs. In the current study, an intelligent middleware platform is proposed to manage and utilize a heterogeneous private edge cloud system infrastructure efficiently. The new platform consists of two layers: SDN and a machine learning-based multi-network management layer and a machine learning-based resource management layer. The multi-network management layer aims to use the capabilities of machine learning and SDN to enable efficient and reliable communication among devices interconnected via multiple heterogeneous mobile ad hoc and infrastructure-based LANs. The resource management layer aims to leverage regression analysis and reinforcement learning methods for efficiently allocating and managing heterogeneous computing and network resources. The platform aims to support smart city and 5G network applications with diverse QoS and system resource requirements. This study also discusses the challenges involved in the design of a middleware platform for complex heterogeneous systems.

Our future objective is the development and performance analysis of a software-defined network and machine learning-based multi-network management layer and machine learning-based resource management layer. This includes the development of several network and resource management algorithms and protocols such as multi-network discovery and monitoring protocol, multi-network routing protocol, and machine learning-based multi-network aware resource allocation algorithms.

## Figures and Tables

**Table 1:** Latency and data rate requirements of smart city and 5G use cases [5,6,7,8].

Use Case	Latency	Data Rate
Factory Automation	0.25–10 ms	1 Mbps
Process Automation	50–100 ms	0.1–100 Mbps
Intelligent Transport Systems	10–100 ms	10–700 Mbps
Robotics and Telepresence	1 ms	100 Mbps
Virtual and Augmented Reality	1 ms	1 Gbps
Health Care	1–10 ms	100 Mbps
Serious Gaming	1 ms	1 Gbps
Smart Grid	1–20 ms	10–1500 Kbps
Education and Culture	5–10 ms	1 Gbps

**Table 2:** A feature-wise comparison of relevant resource allocation and offloading schemes.

	Mobile Cloud	Edge Cloud	Mobile Ad Hoc Cloud	Heterogeneous Network	Real-Time	Parallel Transmission	Network Aware	Link Lifetime Aware	Energy Aware
[2]	✕	✕	✓	✕	✓	✕	✓	✓	✓
[33]	✕	✓	✕	✕	✓	✕	✕	✕	✕
[34]	✓	✕	✕	✕	✓	✕	✕	✕	✕
[62]	✕	✕	✓	✓	✓	✕	✕	✕	✕
[63]	✕	✕	✓	✓	✕	✕	✕	✕	✓
[64]	✓	✕	✕	✕	✕	✕	✓	✕	✓
[65]	✕	✓	✕	✕	✕	✕	✓	✕	✕
[66]	✓	✕	✕	✕	✕	✕	✓	✕	✓
[67]	✓	✕	✕	✓	✕	✕	✓	✕	✓
[68]	✕	✓	✕	✕	✓	✕	✓	✕	✓
[69]	✕	✕	✓	✕	✕	✕	✕	✕	✓
[70]	✕	✕	✓	✕	✕	✕	✓	✕	✓
[71]	✕	✕	✓	✕	✕	✕	✕	✕	✓
[72]	✕	✕	✓	✕	✕	✕	✓	✕	✓
[73]	✕	✕	✓	✕	✕	✕	✓	✕	✕
[74]	✕	✕	✓	✓	✕	✕	✓	✕	✕
[75]	✕	✕	✓	✓	✕	✕	✓	✕	✕
[76]	✕	✕	✓	✕	✕	✕	✓	✓	✓

**Table 3:** Summary of existing middleware platforms.

	Existing Middleware Platforms for Mobile Computing Systems	Proposed Middleware Platform for a Heterogeneous Private Edge Cloud System
Multi-network aware	**✓**	**✓**
Efficient utilization of multi-network environment	***×***	**✓**
Complex multi-network aware (a complex multi-network infrastructure that integrates ad hoc and infrastructure-based network technologies)	***×***	**✓**
Sensing or actuation services	**✓**	**✓**
Computing and storage services	**✓**	**✓**
Computing, storage, sensing, and actuation services	***×***	**✓**
Utilization of heterogeneous high-end devices accessible via wireless ad hoc networks	***×***	**✓**
Mobility management	**✓**	**✓**
Failure management	**✓**	**✓**
Link quality and lifetime aware	**✓**	**✓**
Energy-aware	**✓**	**✓**
Link quality, link lifetime, and energy-aware	***×***	**✓**

## Data Availability

Not applicable.

## Glossary of Acronyms

**Acronyms**	**Description**
IoT	Internet of things
LTE	Long-term evolution
5G	5th generation mobile network
SDN	Software-defined network
LAN	Local area network
PEC	Private edge cloud
MAC	Mobile ad hoc cloud
MANET	Mobile ad hoc network
QoS	Quality of service
MQTT	Message queuing telemetry transport
HTTP	Hypertext transfer protocol
RPi	Raspberry Pi
BLE	Bluetooth low energy
DSRC	Dedicated short-range communications
